# Unveiling Nanocellulose from the Perspective of Morphological Engineering

**DOI:** 10.3390/molecules31162743

**Published:** 2026-08-07

**Authors:** Qian Zhang, Zitian Ding, Xin Wang, Jintian Huang

**Affiliations:** College of Material Science and Art Design, Inner Mongolia Agricultural University, Hohhot 010018, China; zhangqian19970123@163.com (Q.Z.); dzt17866513499@163.com (Z.D.)

**Keywords:** nanocellulose, morphology, control, application

## Abstract

Green bioeconomy materials supported by biomass resources will undoubtedly be the next economic form. Nanocellulose is a carbon-containing renewable energy source that can replace fossil energy, and it can help alleviate the current dual pressure of environmental deterioration and energy shortage. With the rapid growth of nanocellulose in various application fields, the problem that different types of nanocellulose are better suited for different applications has emerged. This article starts with the morphological engineering of nanocellulose and establishes a line from the preparation method as the starting point to the application as the end point. When the starting point is the same, turning on different switches along the line will result in cellulose presenting different morphologies, thus corresponding to different endpoints. Our aim is to deeply explore the influence of this “regulation” in the preparation process on morphology, to discuss the inevitable relationship between different morphologies and applications, and to establish a causal relationship between different factors in a progressive manner. We hope to further the in-depth research on nanocellulose by deepening the understanding of the regulation and control strategies of the three-level structure of cellulose at the nanoscale and the design of functional characteristics.

## 1. Introduction

At present, materials used in human daily life cause increasingly serious problems such as the consumption of non-renewable resources and pollution to the ecological environment, making the use of biomass resources to prepare new materials more and more urgent [1]. Biomass resources refer to organisms produced by photosynthesis [2]. This biomass resource has the advantages of low cost and environmental friendliness and can solve the current dual pressure in terms of environmental degradation and energy shortage [3]. However, realizing the development and utilization of this natural resource and the energy-efficient, high-value and integrated utilization of agricultural and forestry biomass is extremely urgent [4].

Cellulose, as a bio-based material, provides almost unlimited resources for functional composites and, as a sustainable technical material, can meet global challenges [5]. In recent years, cellulose in a nanostructured form, namely nanocellulose, has proven to be one of the most prominent green bioeconomic materials in modern times [6]. Microfibrillated cellulose was first mentioned in 1983, and the method of obtaining nanocellulose through surface stretching and mechanical treatment continues to this day [7]. Most preparations of nanocellulose use top-down or bottom-up preparation methods [8]. Differences in preparation methods can achieve surface modification and morphology control of cellulose. During the processing process, when the hierarchical structure of cellulose is destroyed, there is no doubt that the change in the morphological structure is given priority. When the size of a material reaches the nanometer range, the material characteristics are not only related to the chemical structure, but also affected by the shape and size [9]. So far, there has been no comprehensive summary and comparison on the shape and structure of nanocellulose. There is no doubt that the size and morphology of nanocellulose have a significant impact on performance [10,11]. For example, nanocellulose fibers with high length-diameter ratios and bacterial cellulose are more suitable for research on flexibility experiments [12]. However, nanocellulose with shorter size has higher hydrophobicity, which is conducive to reducing the interfacial tension between oil and water, can produce higher coverage of oily droplets and can obtain better results when preparing emulsions [13]. Even when model predictions are used, the morphology of nanocellulose is an important factor that cannot be ignored [14,15,16,17]. This is foreseeable, and the impact of morphology changes on properties (such as the size of the crystallization area) determines performance. Shape control is the key to enriching nanotechnology applications. In-depth understanding of the internal morphology formation mechanism of nanocellulose and control structural properties are important ways to improve the application scenarios of nanocellulose.

In recent years, nanocellulose has been the subject of extensive research, but it has typically been addressed only as a minor component within broader reviews. Currently, most research on nanocellulose is application-oriented, with very little attention paid to fundamental issues. So far, the classification of nanocellulose based on morphological structure and its impact on various applications has not attracted attention. When the preparation method is the same, nanocellulose with different shapes can be obtained by controlling different factors. Through a review of this method, not only can the application range of nanocellulose be expanded, but also the understanding of the structure of nanocellulose can be further deepened. Therefore, this paper first introduces and then adjusts the influence on morphology through preparation methods. The more suitable applications of spherical (SNC), rod-like (CNC) and fibrillar (CNF) nanocellulose are reviewed. and the decisive factor that the topography can be in this field. Our overall goal is to explore the relationship between preparation methods–morphology–application, promote an in-depth understanding of basic research on nanocellulose, and achieve tailor-made morphology and structural design for subsequent applications of nanocellulose.

## 2. Origin and Structural Characteristics of Cellulose

Cellulose is a linear polysaccharide composed of β-D-glucopyranose units linked by β-1,4-glycosidic bonds. It is a semi-crystalline polymer consisting of both highly ordered crystalline regions and less ordered amorphous regions. The crystalline regions are stabilized by extensive intra- and intermolecular hydrogen bonds. In contrast, the molecular arrangement in the amorphous region is relatively loose. This region is more susceptible to contact with chemical reagents [18]. There is no distinct transition between the crystalline and amorphous regions [19,20].

### 2.1. Source of Cellulose

The material sources of cellulose in nature encompass plant biomass (e.g., wood and agricultural residues), algae, fungi, bacteria, and animal sources (e.g., tunicates). The following discussion is limited to plant-based cellulose sources. Woody biomass can be divided into wood and roots, while agricultural biomass comprises leaf, seed, core, grass, and bast fibers [21,22]. Cellulose is synthesized in these plants by cellulose synthase complexes located on the plasma membrane. Therefore, since nanocellulose is derived from living organisms, research on it requires interdisciplinary knowledge [23]. Since the beginning of the 18th century, various physical, chemical, and combined methods have been developed for cellulose extraction from the above raw materials [24].

### 2.2. Structure of Cellulose

Under unlimited amounts of raw materials and extraction methods, the most common forms of crystalline cellulose obtained are types I and II. Changes in the crystal structure are mainly affected by the intra-and intermolecular hydrogen bonds, with the bond angles and lengths of the glucose-based rings not significantly different for the different crystal forms. However, the angles between the base rings and the side chain and base ring can rotate significantly, leading to changes in the crystal structure. In general cellulose I and cellulose II have been most commonly used and studied. Such changes are generally confirmed by X-ray diffraction (XRD), which is a well-established method for identifying partially crystalline materials and is often used to determine the crystal structure of polymers such as cellulose for the first time [25,26]. XRD can also be used to calculate the crystallinity of cellulose, which reflects the ratio of crystalline to amorphous regions. Crystallinity directly affects cellulose’s mechanical properties, chemical stability, and reactivity. The crystallinity of untreated cellulose is generally distributed over 65–85% [27] according to XRD [28] and has been obtained along with crystal size [29] and interplanar spacing [30]. Iα and Iβ are also present in different proportions as allomorphs in the fibrils of type I cellulose. Bacterial cellulose is dominated by Iα and plants by Iβ and can also be calculated from the intercrystalline spacings obtained by X-ray diffraction [31]. The FTIR method and other testing methods, such as Raman spectroscopy and nuclear magnetic resonance (NMR) spectroscopy, have also been used to ascertain the different crystal structures in cellulose, resulting in peak shifts. In particular, XRD is primarily used in cellulose research for crystal structure and phase identification, as well as for determining crystallinity and grain size. NMR spectroscopy, on the other hand, can provide in-depth insights into the chain conformation, hydrogen-bonding patterns, and local chemical environment of cellulose at the molecular level. The combined use of these two techniques allows for a comprehensive understanding of the structural characteristics of nanocellulose. The increase in characterization methods has been accompanied by the enhanced application of computer calculations to study the structural model of cellulose and has resulted in the proposal of extended-chain, bent chain, and bent and twisted chain models for the crystal structure of cellulose [32,33]. The extended-chain model was the first cellulose molecular-chain model developed and is currently used to simulate and analyze the structure, crystal transformation, dissolution and swelling, chemical modification, and composite applications of cellulose.

## 3. Effect of Preparation Method Control on Morphology

### 3.1. Acid Hydrolysis

In 1947, Nickerson et al. [34] began to explore the hydrolysis process of cellulose by hydrochloric acid and sulfuric acid. When the acid is diluted with deionized water, a large amount of H^+^ will be produced to act on the amorphous region of cellulose (preferential), which will interact with the oxygen atom on the glycosidic bond between two glucose units in cellulose, protonating it and forming the corresponding conjugated acid, and the C-O bond of the conjugated acid is broken to form a cyclic carbocation. Water molecules react with cyclic carbocations and release H^+^. H^+^ ions preferentially attack the amorphous regions of cellulose, where the structure is relatively loose. Therefore, the degree of hydrolysis depends on the relative proportion of amorphous and crystalline regions in the cellulose feedstock. The cellulose molecular chain is broken, hydrolyzing the cellulose to the nanometer level. Currently, acid hydrolysis is one of the most common methods to prepare nanocellulose [35,36], and it is also the most frequently reviewed. Nanocellulose with different morphologies can be prepared by adjusting influencing factors such as acid type, reaction temperature and time, acid concentration, solid-liquid ratio and cellulose source [37,38]. The most commonly obtained nanocellulose morphologies using this method are SNCs, CNCs, and CNF. And the acid hydrolysis method degrades the non-crystalline areas of cellulose; the crystalline areas and a small amount of non-crystalline areas are left behind. Therefore, obtaining CNCs under this method has natural advantages [39]. Generally, they have a more closely arranged microscopic morphology [40]. When using this method, by selecting different raw materials, it is possible to achieve the transformation from CNCs to CNF. The cellulose in the stalks and phloem of Apocynum venetum is a typical example. CNF with a longer length and thin diameter network structure can be separated from the stalks of apocynum venetum, while short nanostructure CNCs with a rod-shaped structure can be separated from the phloem [41]. This different morphology is less affected by the original shape of the raw material. The main influencing factor is differences in cell distribution. The phloem contains long, well-developed fibers, whereas the fibers in the straw are shorter. The preparation of CNF by acid hydrolysis is uncommon because the reaction is violent and usually results in CNCs. However, when straw is used as the feedstock, a certain proportion of lignin and hemicellulose remains after pretreatment [42]. During acid hydrolysis, these residues can modulate the reaction intensity and partially protect the cellulose structure, thereby preserving its fibrous properties.

Sulfuric acid (H_2_SO_4_), hydrochloric acid (HCl), nitric acid (HNO_3_), etc., can hydrolyze cellulose. The main role is played by H^+^, which plays a consistent role in these processes. The difference is that it contains anions. When hydrolyzed with H_2_SO_4_, nanocellulose has better stability and dispersibility. This is because when H_2_SO_4_ is used for hydrolysis, the hydroxyl groups in the nanocellulose undergo sulfonation and esterification reactions with H_2_SO_4_, introducing sulfonic acid groups and sulfate esters. The formation of these two types of groups helps reduce the zeta potential of nanocellulose suspensions and improve dispersion and stability [43]. However, thermal stability will be reduced. Hydrolysis using HNO_3_ will introduce additional carboxyl and aldehyde groups. However, hydrolysis with HCl does not introduce additional groups. Therefore, thermal stability will not be reduced. When the raw materials are the same, the shape of nanocellulose depends on the type of acid. Mahmud M M et al. [44] found that when hydrolyzed for 1 h at 50 °C using 50% sulfuric acid or 37% hydrochloric acid, the resulting cellulose is in the form of CNCs. However, when hydrolyzed for 12, 24, or 48 h at 50 °C using 85% phosphoric acid, SNCs are formed, and the longer the hydrolysis time, the smaller the particle size. The spherical formation of phosphoric acid may be caused by similar electrostatic interactions. The most speculated reason why SNCs were successfully extracted is that some amorphous areas in cellulose are more susceptible to gradual hydrolysis by hydronium ions than crystalline areas. The morphology first appears as rod-like cellulose with different lengths. When the hydrolysis process can continue, the rod-like cellulose is further hydrolyzed into irregular nanoparticles with smaller morphology. Affected by van der Waals forces, the particles are stacked together, and the hydroxyl groups contained in the short-chain cellulose on the surface undergo hydrogen-bonding self-assembly, thereby forming a spherical structure. The location of the cellulose chain break determines the reactivity of SNCs [45]. Another hypothesis is that the ionic strength of phosphoric acid may also play a role in determining the shape of the cellulose produced. This needs further exploration. At present, research on SNCs is mainly focused on their preparation, while their generation mechanism and application forms still require further investigation.

Conventionally, acid concentrations for processing cellulose into nanocellulose include 55–65% sulfuric acid solution and 8–12% hydrochloric acid solution. In recent years, the regulation of acid concentration has been a key area of research. However, the use of acid is not limited to these conventional concentrations. Nanocellulose with different morphologies obtained by controlling the concentration is currently only concentrated in CNCs. Cellulose extracted from empty oil palm fruit bunches was hydrolyzed for 3.5 h in a 97% sulfuric acid solution at a solid-to-liquid ratio of 1:80 and 50 °C, resulting in a crystallinity of up to 96% for CNCs [46]. This value may be affected by the XRD calculation method. When the acid concentration is low (35–65%), the size will increase to varying degrees as the concentration decreases [47]. But it will not increase to the micron level, that is, it will not form a fibrillar shape. When conventional dilute acid (e.g., 5%) is used, it is often difficult to obtain B-CNC or nanocellulose without additional treatments. However, recent studies have shown that when dilute acids are mixed with oxidizing agents (such as H_2_O_2_) in a single reaction system, nanocellulose can be effectively isolated even at low acid concentrations (e.g., approximately 3% H_2_SO_4_) [48]. Increasing the reaction time can produce effects similar to increasing the acid concentration. Notably, extremely low acid hydrolysis has also been explored; however, the product in some early reports was microcrystalline cellulose (MCC) rather than CNCs [49]. The CNCs separated by sulfuric acid hydrolysis will decrease in size and yield as time increases [50]. However, the crystallinity of CNCs increases with the increase in hydrolysis time [51]. This result further proves that CNCs originate from the crystallization region of cellulose. However, the effect of acid concentration on other morphologies has been rarely studied.

The difference in the degree of acidification can be controlled by acid concentration and acid type. But it is difficult to control for a single factor. Noguchi Y [52] used phosphorylation and urea treatment to obtain CNF. This is a method that requires control of the degree of phosphorylation. It also takes measures to increase the charge by chemical modification to promote the separation of microfibers from each other into CNF. The role of urea is to act as a spacer to prevent cross-linking between cellulose microfibers. In addition to limitations on methods, the selection of raw materials is also harsh. In addition, controlling the acid hydrolysis time is a simpler method. There is an approximately linear relationship between the size of the prepared SNCs and the processing time. As the hydrolysis time increases (with higher acidification), the nanocellulose size decreases. This indicates that the obtained SNCs are spherical due to the shortening of the nanocrystals due to hydrolysis of the amorphous region. Longer hydrolysis treatments lead to the disappearance of more amorphous areas. This may be due to the complete removal of amorphous regions in the cellulosic material with increasing hydrolysis time, confirming the high resistance of crystalline regions in the SNC structure to acid hydrolysis [53]. Finally, the cellulose crystallinity index increased with the decrease in particle size.

Inspired by the fact that CNCs originate from the cellulose crystallization region, the source of SNCs will be affected by the crystal structure. Cellulose I has strong polymorphic transformation ability and can be converted from cellulose I to cellulose II through one of two processes: regeneration or mercerization [54]. Before acid treatment, the amorphous region of cellulose is generally swollen by these two methods, resulting in expansion of the cellulose II structure, an increase in chain spacing and structural disintegration. This effectively changed the morphology of nanocellulose from irregular shapes to spherical cellulose nanocrystals [55]. Therefore, Type II SNCs are easier to obtain. Another theory is that the amorphous regions of cellulose II are more prominent than cellulose I. Due to the uniform distribution of cellulose II crystals, the SNC size is more uniform [56].

### 3.2. Oxidation Method

The most common method for preparing nanocellulose by oxidation is treatment with sodium periodate or 2,2,6,6-Tetramethylpiperidine-1-oxyl (TEMPO). This is a method completely different from acid hydrolysis. The difference is that periodate oxidation can oxidize the C2 and C3 hydroxyl groups of the glucose unit into aldehyde groups under mild conditions, breaking the C2-C3 carbon bond. TEMPO-mediated oxidation selectively converts the primary hydroxyl group at the C6 position into a carboxyl group, preserving the molecular backbone of cellulose and thereby maintaining the fibrous morphology. In contrast, neutral nanocellulose retains its native hydroxyl groups, whereas oxidation modification introduces groups such as carboxyl and aldehyde groups.

This method mainly oxidizes TEMPO into nitrosyl ions. Nitrosyl ions can selectively oxidize primary alcohol hydroxyl groups on the surface of natural cellulose microfibers, thereby introducing carboxyl groups and aldehyde groups. Because it maintains the characteristics of type I cellulose and has a highly crystalline area, it is conducive to maintaining mechanical properties [57]. Typically, CNF is obtained. During the preparation of CNF, the content of carboxyl groups is a major factor in judging the oxidation degree of CNF. It is affected by reaction time [58], pH value (which also affects the degree of polymerization, yield, group content, and brightness of CNF) [59], mechanical treatment methods, etc. [60]. Using a highly efficient catalyst, the carboxyl content can exceed 1.1 mmol/g within 1–4 h of oxidation, and the CNF yield can reach over 56% [58]. In the reaction, the morphology can be controlled by controlling the amount of aldehyde contained. Due to the high accessibility of the amorphous region of cellulose, the oxidation reaction mainly proceeds in the amorphous region and then gradually enters the crystalline region. With the increase in aldehyde content and other factors, as the oxidation reaction progresses, the higher reactivity of the amorphous region and the break of the molecular chain accompanying the oxidation reaction explain the formation of CNCs at low oxidation levels. As the aldehyde content increased from 450 μmol/g to 1480 μmol/g, the average particle size of the product gradually decreased from 890 nm to 80 nm [61]. As the size of nanocellulose gradually decreases, the transformation from CNF to CNCs is achieved. This process is more or less similar to the acid hydrolysis process used to form CNCs and also results in a similar size range. But it has better heat resistance due to hemiacetal connection. It also exhibits a lower intrinsic viscosity than CNCs obtained by acid hydrolysis. Its final decomposition temperature can reach 450 °C (compared to 305 °C for conventional nanocellulose), and its intrinsic viscosity is only one-fourth that of conventional nanocellulose [62].

The most important factor in the transition from CNF to CNCs or SNCs using this method is the control of the degree of oxidation. As the degree of oxidation increases, the length of the cellulose chains gradually decreases, and the aspect ratio decreases accordingly [63]. When transitioning to CNCs, the degree of oxidation can be controlled by factors such as temperature, pH, concentration and reaction time during the process. However, under general circumstances, it is less affected by raw materials [64]. SNCs can also be obtained through such methods. By increasing the degree of oxidation, the cellulose chain can be further peeled off. The morphology changes from crystal strings of beads to hollow microspheres [65].

### 3.3. Ionic Liquid Method

Concentrated sulfuric acid hydrolysis is one of the earliest and most widely used methods to prepare nanocellulose, but the recovery of waste acid is difficult. Ionic liquids (ILs) are a new type of green solvent or swelling agent that is considered an ideal substitute for separating cellulose nanowhiskers from cellulose matrices under less harsh conditions [66]. The dissolution mechanism of ionic liquids primarily involves the formation of hydrogen bonds between the anions in the liquid and the protons on the hydroxyl groups of cellulose, while the cations interact with the oxygen atoms in the glycosidic bonds, causing the hydrogen bond networks within and between cellulose molecules to break and inducing the transition of cellulose from a solid to a liquid state. Ionic liquids are molten salts that have excellent potential for the extraction, utilization and transformation of cellulose [67,68]. Ionic liquids can reduce the shear force on cellulose, disrupt the cellulose hydrogen-bonding network, fully penetrate into the gaps of cellulose crystals and promote defibrillation. Unlike acid hydrolysis and oxidation, the ionic liquid method involves the dissolution of cellulose. Changes in properties such as the size and morphology of cellulose depend on several parameters, including the cellulose source, hydrolysis conditions, and post-treatment techniques such as homogenization and sonication [69]. However, there are few studies on these factors.

ILs anions tend to actively interact with cellulose sites and water molecules during dissolution. Thus, the cation type of ILs plays a decisive role in the particle size and morphology of cellulose. Cellulose treated with [PMIM][Cl] (containing propyl substituents) exhibits smaller particle sizes (approximately 20–100 nm) and a narrower size distribution; in contrast, cellulose treated with [EMIM][Cl] (containing ethyl substituents) tends to form agglomerates (particle sizes of 480–1000 nm) [70]. The slower the phase separation, the easier it is for SNCs to be formed. Ionic liquids with an odd number of alkyl chains on the cationic end are more effective than those with an even number of alkyl chains in the preparation of CNCs. However, when preparing CNCs by this method, particle aggregation and relatively large particle size have not yet been solved due to lack of process optimization [71]. Therefore, it is usually combined with other methods using a two-step process, such as ionic liquid-assisted low-concentration acid treatment. This allows for a further reduction in size while significantly lowering the acid concentration [72].

It is also possible to prepare CNF using this method. This process mainly destroys the hydroxyl group in the cellulose molecule, breaks it, and applies mechanical force to defibrillation to obtain CNF with a nanofiber network structure [73]. To realize the transition from CNF to CNCs, it can be achieved by controlling the proportion of ILs. As the IL proportion increases, the degree of effect increases, and CNCs can be generated [74].

### 3.4. Deep Eutectic Solvent System

Deep eutectic solvent (DES) is a new type of solvent similar to ionic liquids. It is also a multi-component mixture composed of hydrogen bond acceptors such as quaternary ammonium salts and hydrogen bond donors of polyols, carboxylic acids, etc. It was first discovered in 2003 by studying a eutectic mixture of urea and a series of quaternary ammonium salts [75]. It not only retains the advantages of ionic liquids such as strong solubility and low vapor pressure, but also has the unique advantages of simple synthesis, low cost, and little influence by water.

Recently, a new class of methods for preparing CNF with ultra-fine and long lengths that can be used for industrialization has been mentioned. This type of CNF has a diameter of 3.4 nm and an aspect ratio of as high as 2500. And at high concentrations, CNF suspensions also have excellent stability and are easy to store, transport, process and utilize [76]. The longer aspect ratio allows for better utilization in 1D filaments, 2D films and 3D aerogels [77]. This is a method that needs to be used in conjunction with mechanical processing under normal circumstances, which is common. It is difficult to obtain CNF by simply using ultrasonic treatment or using DES alone. This can be expected. In addition to removing other impurities such as lignin and hemicellulose, DES can also increase the accessibility of remaining cellulose. The degree of polymerization and viscosity are also reduced, which is more conducive to fibrillations during subsequent mechanical treatment. The morphology can be affected by controlling the concentration of DES. Generally, as the proportion increases, the size decreases, transitioning from CNF to CNCs. The properties of CNCs itself are also affected by the concentration of DES. As the concentration increases, the diameter size becomes smaller [78].

### 3.5. Other Methods for Preparing Nanocellulose with Different Morphologies

#### 3.5.1. Lewis Acid Method

The ordinary one-pot method increases the difficulty of acid recovery and cannot reach the concentration of cellulose hydrolysis. In addition, due to the interaction of multiple substances, the reaction process cannot be fully monitored and adjusted. Therefore, the development of a green, environmentally friendly, innovative and efficient method for preparing nanocellulose has gradually attracted the attention of scholars. The amorphous region of cellulose is destroyed under mild conditions to prepare nanocellulose.

Lewis acid treatment is a method that can maintain the crystalline form I of nanofibrillated cellulose and improve the crystallinity of nanofibrillated cellulose by hydrolyzing amorphous regions. CNCs prepared by this method have relatively high thermal stability [79]. SNCs can be prepared by excessive hydrolysis of the cellulose crystallization zone caused by the co-presence of acid and solvent. Due to the competitive hydrogen bond formation between Lewis acid and cellulose -OH groups, the strong hydrogen bond interaction in cellulose is weakened. As time increases, the number of hydrogen bonds broken in cellulose increases, and accordingly more coordination bonds can be distributed. When the destruction of hydrogen bonds in solution and the formation of coordination bonds reach an equilibrium, the strength of the original hydrogen bonds in cellulose molecules is the lowest, forming SNCs [80]. The hydrolysis conditions and effects of Lewis acid can be quite severe, but unlike the above, this method can maintain the crystallinity index of SNCs [81].

#### 3.5.2. Enzymatic Hydrolysis Method

The method is greener and environmentally friendly. The cellulase hydrolysis process involves the simultaneous synergistic action of multiple different enzymes (complex enzymes). Unlike the acid hydrolysis method, these enzymes interact to randomly destroy the β-1,4-glycosidic bond. Exoglucanases mainly act on the linear end of the cellulose molecule, destroying the crystalline region of cellulose [82].

Nanocellulose with different morphologies can be obtained using this method. This is similar to acid hydrolysis, where the enzyme preferentially attacks defective parts of cellulose, thereby retaining a highly ordered and relatively stable crystalline part, with a structure of crystal form I [83]. It can also be used to prepare CNCs [15]. SNCs can be obtained when the degree of enzymatic hydrolysis reaction is enhanced, such as using a synergistic effect of complex enzymes or increasing the concentration of enzymes [39]. With the increase in the degree of enzymatic hydrolysis reaction, the morphology of nanocellulose can transition from strip-shaped nanocellulose to smaller single SNCs after the coexistence of SNCs and strip-shaped nanocellulose [84].

#### 3.5.3. Mechanical Method

Mechanical methods mainly rely on controllable mechanical external forces generated by various machines to destroy the internal structure of cellulose at the physical level, making the size reach the nanometer level. Mechanical treatments may include ball milling [85], ultrasonic crushing [86], high-pressure homogenization [87], steam explosion [88], cryocrushing [89], microfluidization and grinding [90].

Mechanical methods are mainly used for the preparation of CNF. Compared with microfluidization and homogenization, less refining pretreatment steps are required when preparing CNF with micromills [91]. This means that this method may be more direct and cause less damage to the fiber structure, which helps preserve the high aspect ratio of CNF. However, the principle for preparing CNF is similar. It is produced by overcoming intermolecular and intramolecular forces under severe forces and penetrating from the amorphous region to the partially crystalline region. Among these methods, high-pressure homogenization is the most commonly used method. In high-pressure homogenization, the degree of fiberization is affected by the pressure applied by the homogenization and the number of cycles. The degree of cellulose fibrillation depends on the number of homogenization cycles and the pressure applied. Therefore, the aspect ratio of CNF is more controllable. In grinding, the degree of cellulose fibrillation depends on the number of passes through the mill, the distance between the disks, and the shape of the disk channels. It enables size control ranging from the micrometer to the nanometer scale.

The preparation of SNCs involves inducing cellulose to peel into nanometer-size structures through the combined action of micropolarity and mechanical force in the external environment and different crystal faces of cellulose. The fiber’s shape and size are changed by changing the external environment and irreversible changes caused by external mechanical forces to improve the bonding potential of the fiber. It is worth noting that when only processed mechanically, better results may be achieved with undried cellulose. Because hydrogen bonds can be regenerated between celluloses when dried, undried pulp more easily undergoes micro-nano-fiberization [92]. In most cases, when only mechanical external force is used to act on cellulose, in addition to requiring a large amount of energy loss, it is generally difficult to meet the conditions. Therefore, physical or chemical treatment means are needed for pretreatment before external mechanical treatment. Under the condition of adding organic dispersants, reactive reagents and auxiliary agents, swelling in the fibrils is promoted, and then the hydrogen bonds of cellulose molecules are broken through mechanical treatment. The chemical reaction on the cellulose surface and mechanical crushing synergistically achieve the preparation of SNCs [93,94,95].

## 4. Application of Rod-like Nanocellulose

Due to growing environmental issues and increasing requirements for material use, more and more attention has been focused on the development of nanocellulose-based composites or nanocellulose functional materials [96]. However, different forms of materials have different properties and different uses [97,98,99], and the properties of composite materials are influenced by the morphology, size, and crystallinity of the cellulose material [100]. Due to its high crystallinity, moderate aspect ratio, and rigid structure, CNCs are particularly suitable for use as a reinforcing material, for emulsion stabilization, and for liquid crystal formation; CNF, with its micro- and nanoscale dimensions and flexible network structure, is an ideal choice for the manufacture of transparent films, electronic devices, and dispersion systems; SNCs, due to their large specific surface area, high symmetry, and isotropic properties, perform exceptionally well in adsorption, antibacterial, and membrane applications. Understanding the relationship between these structures and their properties is crucial for selecting the appropriate nanocellulose morphology for specific applications [101].

### 4.1. Enhancement

Enhancement is currently one of the most commonly used functions for CNCs [102]. Yuan et al. [103] used CNCs to enforce natural rubber with remarkable results, owing to the geometric characteristics of the CNCs. Oun et al. [104] compounded CNCs with carboxymethyl cellulose, resulting in a 102% increase in the tensile strength and 228% in the elastic modulus for a CNC content of only 10 wt%. This result indicates that, as reinforcing materials, CNCs can significantly improve the mechanical properties of composite materials, such as tolerance [105]. This is because CNCs retain the crystalline zone and therefore have higher stiffness (150 GPa) and strength (7 GPa) [106]. Secondly, although CNCs have a smaller aspect ratio than CNF, they can also form a network structure, which is advantageous for the reinforcement of matrix materials [107]. In addition, CNCs are suitable for use as reinforcing materials because they can form good dispersions, and a good dispersion state is crucial when using CNCs as polymer reinforcement. This is highly dependent on the geometry of the nanocellulose [108].

### 4.2. Emulsion

The strong adhesion of CNC to the interface confers high stability to an emulsion. This is attributed to the unique behavior of CNCs, namely, their rod-like morphology and low surface charge density. CNCs are both effective and stable emulsifiers that significantly reduce the size of oil droplets [109], while CNFs have been shown to do the opposite because CNFs with a large aspect ratio form a high anionic charge during treatment, which is unfavorable for the preparation of emulsions. The morphology and properties of CNCs are thus more suitable for emulsion formation because the specific morphology of these particles enables connection at the interface and the formation of bridge-like structures [110]. A smaller aspect ratio is more conducive to the adsorption of CNCs at the oil–water interface. When the aspect ratio increases, a large steric hindrance is generated behind the oil–water interface, hindering the adsorption of some particles. Secondly, the charge density should not be excessively high. A high charge density produces excessive electrostatic repulsion, which hinders the adsorption of nanocellulose at the oil–water interface. However, CNCs have a lower charge density than CNF, meaning that the electrostatic repulsion between the nanocellulose molecules can be appropriately reduced. These points suggest that the lower aspect ratios and surface charge densities of SNCs render them more suitable than CNCs for this purpose. However, this type of nanocellulose has not been widely used because of instability in its preparation. This is an urgent problem.

### 4.3. Antibacterial Properties

Nanocellulose is also a raw material used in the preparation of natural and environmentally friendly antibacterial agents [111]. Owing to the unique morphology of CNCs, their one-dimensional size and rod-like morphology can induce specific biointerface behaviors in asymmetric structures, improving their antibacterial ability and enhancing their endocytosis and retention [112]. Needle-like CNCs are also of great use. The toxicity of CNCs is entirely dependent on the physical membrane stress caused by the contact of cells with their needle-like structures. This unique morphology gives CNCs the ability to penetrate cell membranes [113].

### 4.4. Liquid Crystals

Liquid crystals are materials that are disordered in their molecular arrangement but maintain the order of orientation [114]. Currently, this approach is applicable only to CNCs studies and is primarily dependent on the geometry. Second, the presence of negative charges on the surface renders the CNC suspension highly stable. Surface charge is key to the formation of cholesteric liquid crystals. When CNCs reach a certain concentration, they spontaneously assemble into cholesteric liquid crystals by evaporation and exist only in the form of a membrane, rendering these particles useful in a wide range of applications such as information encryption, optical coding, and optical data storage.

## 5. Application of Fibrillar Nanocellulose

CNFs are the most widely used class of cellulose. The final product prepared using CNF as a raw material has a variety of applications owing to its unique chemical and physical properties together with superior performance. CNFs are typically used as substrates due to basic properties such as high mechanical strength, sufficient transparency, a smooth surface, and the formation of a high anionic charge during treatment, improving it as a dispersant. Some of these technologies have already reached maturity.

### 5.1. Transparent Paper

High transparency is one of the most obvious features of CNF because the size of the nanofibers is much smaller than the wavelength of visible light, meaning that it can attain considerable forward light scattering. The optical transmittance is generally above 80% (at 550 nm) [115]. Good optical properties are essential for thin film preparation, and the resulting films could break the monopoly held by non-renewable plastics [116]. Although the size of CNF is as small as possible, the fibers still have the ability to deflect light in its direction of propagation, resulting in high haze. Haze can be reduced by weakening the light scattering at the cellulose–air interface on the film surface [117].

This high transparency and the large difference between the diffusion transmittance and mirror transmittance are promising for optoelectronic applications. Using CNF as a transparent substrate for optoelectronic materials to overcome synthetic chemical-based optoelectronic materials is a long-term challenge. CNF can negate the loss of material dimensional integrity and optical transparency in high-temperature operating environments due to a low glass transition temperature and high coefficient of thermal expansion when other materials are used as polymer substrates [118], and because of their excellent mechanical properties, CNF can also be used in the construction of ultrathin substrates [119].

### 5.2. Electronic Devices

The development of CNF for use in electronic devices is one of the most rapid applications. The development is so fast that it depends, first, on the various tunable performance parameters of CNF in this form, including the morphology (such as well-dispersed fibrillar and network structures), size (such as width, length, and aspect ratio, which is a typical micro/nanostructure), surface chemistry, and suspension environment [120]. Adaptable properties include a smaller bending radius, smaller coefficient of thermal expansion, lighter mass, and higher mechanical strength with better O_2_ and H_2_O barrier properties, as well as high dielectric properties (dielectric constant ε > 6) and good responsiveness to electric fields. However, these factors do not necessarily imply that the material is better suited for this type of application. The decisive factors are its better binding ability, initial gel-like appearance, and high processability compared with other morphologies. In addition to a more controllable film thickness, a smoother film surface is obtained, and rollability, printability, and functionality can be easily integrated into a substrate or functional layer via casting, filtering, coating, or extrusion and then combined with transistors, solar cells, and sensors [101]. By regulating these parameters, a controlled orientation structure can be produced by using chemical charge and size matching for application in electronic devices, and the problems of poor mechanical properties and processability of the devices have been addressed [121]. The advantage of the highly flexible and directional ion transport is that other substrate materials do not form.

However, development in this field has not yet been achieved. This is a long-standing development process, and changes over time and the progress of science and technology have been observed, with the first being the ability to combine or assemble conductive materials, followed by their application in solid-state electronics, optoelectronics, and flexible/wearable electronics. This led to an even greater increase in the CNF performance requirements. CNF can reversibly and strongly adhere to any surface and detach without leaving any residue [122].

### 5.3. Further Applications

The large aspect ratio, good dispersion, and network structure formed when the produced film is compounded with other materials maintains the mechanical properties of the film, rendering composite film transparent and sufficiently flexible. The most basic performance guarantees the mechanical properties and transparency [123], and the mechanical properties of these particles have led to their wide use in food preservation films [124], multifunctional films [125], and flexible electronic devices [126]. CNFs are usually prepared via chemical modification to increase the charge and promote the separation of microfibers. This characteristic renders CNF research significant for adsorption [127], catalytic degradation [128], oil–water separation [129], smart materials [130], and sustained-release pesticides instead of agricultural films [131]. Owing to their convenient modification, CNFs are usually used to provide new application methods, with the most common, hydrophobic modification, applied in air-filtration membranes [132] and the fabrication of coatings with chemical stability, mechanical durability, and self-cleaning properties. The coatings exhibit significant anti-icing properties and can delay the freezing of water. These particles thus have broad application prospects in industrial fields, such as self-cleaning and anti-icing [133].

## 6. Application of Spherical Nanocellulose

Material properties are determined by structure, which in turn determines the design and application. Emerging nanostructure materials such as SNCs are the focus of cellulose research, with one of the most attractive cellulose transformations being the development of SNCs through various modes [134]. The large specific surface area, low aspect ratio, high symmetry, and other intrinsic properties of SNCs, combined with the quantum effects and surface effects arising from their nanoscale dimensions, endow them with unique advantages in interfacial science, advanced optics, biomedicine, and composite materials—advantages that are not achievable with other morphologies. These distinctive features have established spherical nanocellulose as an important and independent research direction. However, because the preparation of SNCs is imperfect and unstable, their application is still lacking and requires further study, with the stability of the preparation process an important precursor to their wider application.

### 6.1. Adsorption

The most common use for SNCs is associated with their generality and specificity. The universality of biomass is due to its abundant sources and low prices [135]. These special characteristics, which include a large specific surface area, high packing density, higher solution fluidity, and controllable size, render it suitable for adsorption applications, and the morphological properties of a material are important factors in this field. In addition to the SNCs commonly used in this field, other materials with spherical morphologies, such as activated carbon [136], are often used. Of course, it is important to note that it may not be sufficient to rely only on morphological differences to avoid modification of the SNCs. This depends on the contaminant to be treated.

SNCs have a higher binding affinity than other cellulose morphologies and can be used for the adsorption of more heavy metal species [137], together with organic dyes via electrostatic interaction. When used for the adsorption of heavy metals or organic dyes, factors such as the preparation of raw materials lead to differences in the adsorption capacity [138]. In addition, the high accessibility of the SNC hydroxyl (-OH) groups means that it can be easily modified into various materials with various groups for the better adsorption of pollutants with different electrical properties. In addition to heavy metals and organic dye pollutants, the adsorption of pharmaceutical pollutants is an area of concern for environmental protection [139].

### 6.2. Antibacterial Applications

SNCs are commonly used as an antibacterial and biomedical material, with the use of SNCs as a matrix significantly improving the cell adhesion ability of a nanocomposite. Such nanocomposite scaffolds offer potential for biomedical applications, particularly cell adhesion and skin tissue engineering. Biosynthesized nanoparticles have been used for antibacterial and in vitro applications [140]. The unique morphology of SNCs can be used as a model for drug delivery, and in combination with other antibacterial materials, it can be widely applied to both Gram-positive and Gram-negative bacteria, as well as to molds, yeasts, and parasites such as those that cause malaria and toxoplasmosis [141]. SNCs extracted from biomass resources are therefore a key and potentially green nanomaterial for drug and vitamin delivery application [142].

### 6.3. Membrane Materials

Currently, SNCs exhibit good biodegradability, and their most common application is as bio-based membrane material. The low aspect ratio and reduced modulus of dissolved cellulose render the film theoretically devoid of mechanical locking and stiffness properties, resulting in an inevitable decrease in strength. However, SNCs appear to be advantageous for enhancing the mechanical strength of composites when used as a reinforcement [143,144]. This special morphology makes SNCs an effective template for nanohybrids and a high-performance nanocomposite reinforcement. The key factors must therefore be investigated. In addition to improving the mechanical properties of films, the water-vapor barrier properties of SNCs can also be significantly improved and do not affect the smoothness or uniformity of the film surface [145]. These results suggest that nanocomposites that include SNCs, with their high specific surface area and high performance, as reinforcement may be well suited to active food packaging materials. The use of SNCs as a reinforcing agent for the preparation of bionanocomposites has great potential for the development of fully biodegradable food packaging materials [146] which can be used as a functional food packaging material together with hydrophobic or other nano-reinforced food packaging materials [147].

### 6.4. Additional Applications

The large specific surface area of SNCs means that they can also be used in the development of low-cost smart materials for various applications with positive environmental impacts. As a sensor that can sense Cu^2+^ ions, SNCs have a good ability to absorb Cu^2+^ ions [148], and their good uniformity and stability means that they can be used as an additive in Pickering emulsions [149]. The properties of emulsions are affected by the size and morphology of solid particles, and if only the morphology factor is considered, particle association has been shown to positively affect the emulsion stability. Unlike a rod-like phase transition, a spherical shape is more conducive to emulsion formation [150]. Another advantageous application of this unique morphology is its use in bio-nucleating agents, which could overcome major industrial problems such as the slow crystallization of polylactic acid. SNCs can be well dispersed without the need for a surfactant or compatibilizer. Owing to the crystallization ability of the provided material and the interfacial interaction during compounding, the mechanical properties and thermal stability of the composite have been significantly improved [151]. In addition to the aforementioned properties, SNCs can also be used to obtain optical, electrical, magnetic, and thermal properties, with examples including seawater desalination, magneto-optical applications [152], and the use of nanocomposites as polymer electrolyte membranes for lithium-ion battery separators [153]. SNCs exhibit good thermal stability, which is favorable for high-temperature applications [154]. In addition, their application in foods appears to be feasible, with SNCs used as an enhancer in foods where their swelling capacity is higher than that of fibrillar cellulose [155]. The application of SNCs reflects their outstanding contribution to environmental sustainability, and the development of smart materials utilizing the most abundant biopolymers to correct environmental imbalances is the most important application trend in the future [144].

## 7. Conclusions and Prospect

Cellulose is widely studied as a carbon-based renewable energy source that can replace fossil fuels; however, theoretical aspects such as the design of its molecular structure and the precise control of its morphology require further investigation. Regulating cellulose morphology, exploring the relationships among its morphology, structure, and properties, and understanding their impact on applications are current research hotspots, as differences in morphology can broaden the applications of the same material across various fields. Therefore, this paper reviews the classification of nanocellulose morphologies, categorizing them into CNCs, CNF, and SNCs. Emerging nanocellulose morphologies (such as nanosheets and nanoplatelets) are still in the early stages of development; these constitute an important classification system for future nanocellulose morphologies and warrant close attention. The morphology of nanocellulose can be controlled by adjusting its preparation methods, raw materials, and relevant process parameters. Furthermore, establishing a comprehensive understanding of the differences in intrinsic properties (such as crystallinity) among various nanocellulose morphologies is of great significance for the rational design and application of nanocellulose-based materials. Finally, the relationship between nanocellulose morphology and its applications requires further exploration.

## Data Availability

No new data were created or analyzed in this study. Data sharing is not applicable to this article.
